# *Nicandra physalodes* Extract Exerts Antiaging Effects in Multiple Models and Extends the Lifespan of *Caenorhabditis elegans* via DAF-16 and HSF-1

**DOI:** 10.1155/2022/3151071

**Published:** 2022-10-11

**Authors:** Jiqun Wang, Yunyuan Huang, Kaixuan Shi, Lingyuan Bao, Chaojiang Xiao, Tianyue Sun, Zhifan Mao, Jiali Feng, Zelan Hu, Zhenghan Guo, Jing Li, Bei Jiang, Wenwen Liu, Jian Li

**Affiliations:** ^1^State Key Laboratory of Bioreactor Engineering, Shanghai Frontiers Science Center of Optogenetic Techniques for Cell Metabolism, Frontiers Science Center for Materiobiology and Dynamic Chemistry, Shanghai Key Laboratory of New Drug Design, School of Pharmacy, East China University of Science and Technology, Shanghai 200237, China; ^2^Hubei Key Laboratory of Genetic Regulation and Integrative Biology, School of Life Sciences, Central China Normal University, Wuhan, Hubei 430079, China; ^3^Yunnan Key Laboratory of Screening and Research on Anti-Pathogenic Plant Resources from West Yunnan, College of Pharmacy, Dali University, Dali, Yunnan 671000, China; ^4^Key Laboratory of Tropical Biological Resources of Ministry of Education, College of Pharmacy, Hainan University, Haikou, Hainan 570228, China; ^5^Clinical Medicine Scientific and Technical Innovation Center, Shanghai Tenth People's Hospital, Tongji University School of Medicine, Shanghai 200092, China

## Abstract

The development of safe and effective therapeutic interventions is an important issue for delaying aging and reducing the risk of aging-related diseases. Chinese herbal medicines for the treatment of aging and other complex diseases are desired due to their multiple components and targets. Through screening for effects on lifespan of 836 Chinese herbal medicine extracts, *Nicandra physalodes* extract (HL0285) was found to exhibit lifespan extension activity in *Caenorhabditis elegans* (*C. elegans*). In further experiments, HL0285 improved healthspan, enhanced stress resistance, and delayed the progression of neurodegenerative diseases in *C. elegans*. Additionally, it ameliorated senescence in human lung fibroblasts (MRC-5 cells) and reversed liver function damage and reduced senescence marker levels in doxorubicin- (Dox-) induced aging mice. In addition, the longevity effect of HL0285 in *C. elegans* was dependent on the DAF-16 and HSF-1 signaling pathways, as demonstrated by the results of the mutant lifespan, gene level, and GFP level assays. In summary, we discovered that HL0285 had an antiaging effect in *C. elegans*, MRC-5 cells, and Dox-induced aging mice and deserves to be explored in the future studies on antiaging agents.

## 1. Introduction

Aging is characterized by a progressive decline in physiological function, leading to various aging-related diseases, including cancer, neurodegenerative diseases, diabetes mellitus, and cardiovascular diseases, further burdening communities and families [1]. Therefore, delaying aging has become an important issue facing society [2]. Currently, a hot topic for research is the discovery of antiaging agents, which may be promising tools for extending lifespan and improving healthspan. Many antiaging agents have exhibited great potential in retarding senescence. For example, metformin treatment led to a 5.83% mean lifespan extension in male mice [3], and rapamycin treatment prolonged the mean lifespan in male (9%) and female (13%) mice [4]. In addition, new interventions to ablate senescent cells for lifespan extension have emerged, such as D+Q (dasatinib+quercetin) [5, 6] and senolytic vaccination [7]. Although many potential antiaging agents are available, clinical trials and approval for antiaging agents have been rare.

Through various species studied, nine hallmarks associated with healthspan and longevity have been identified [8], suggesting that aging is a complex process mediated by multiple targets and biological pathways. Therefore, achieving an ideal therapeutic effect based on a traditional monotherapy strategy seems to be inadequate and difficult. Compared with molecules targeting a specific target or biological pathway, Chinese herbal medicines exert pharmacological effects through the synergistic action of multiple components and targets, which has significant advantages from an overall perspective. Hence, Chinese herbal medicines have received widespread attention in the treatment of complex diseases, such as aging [9]. For instance, grape seed extract inhibits SASP factors at low concentrations and kills senescent cells at high concentrations, making it a promising antiaging agent [10]. The polymethoxyflavone-rich extract derived from citrus peels can attenuate metabolic syndrome by influencing the gut microbiome and amino acid metabolism and is expected to be an attractive metabolic regulation agent in therapy [11]. The above studies showed that Chinese herbal medicines have great research value and therapeutic potential against aging and deserve to be further explored.

**Figure 1:**
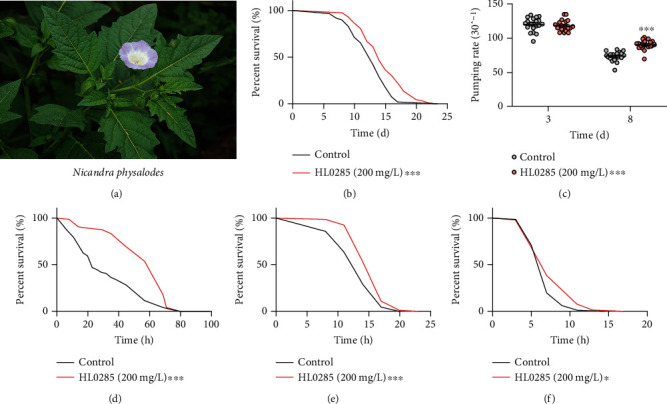
HL0285 extends the lifespan and healthspan of *C. elegans*. (a) Picture of *Nicandra physalodes*. (b) HL0285 (200 mg/L) increased the lifespan of *C. elegans* (wild-type). (c) HL0285 significantly increased the pharyngeal pumping rate of worms on day 8. The effect of HL0285 on stress resistance, including (d) oxidative, (e) heat, and (f) osmotic stress, on day 4. The log-rank (Mantel-Cox) test (b, d–f) and two-way ANOVA along with the Sidak multiple comparisons test (c) were used to calculate the *P* values. The results are presented as mean ± SEM. ^∗^*P* < 0.05 and ^∗∗∗^*P* < 0.001.

With the aim of identifying effective and safe antiaging agents, we screened 836 Chinese herbal medicine extracts through *Caenorhabditis elegans* (*C. elegans*) lifespan experiments [12]. After three rounds of lifespan screening experiments, *Nicandra physalodes* extract (HL0285) (Figure 1(a)) was selected for the study because of its apparent lifespan extension activity. By further study, we revealed that HL0285 could prolong lifespan, enhance healthspan, and delay the progression of aging-related diseases in a *C. elegans* model. In addition, in other models, HL0285 ameliorated the senescence of human fetal lung fibroblasts (MRC-5 cells) and counteracted premature senescence in doxorubicin- (Dox-) induced aging mice. Further mechanistic studies determined that the antiaging effects of HL0285 were dependent on DAF-16 and HSF-1. In summary, HL0285 has great potential in delaying aging and aging-related diseases and deserves to be further explored in studies on antiaging agents.

## 2. Materials and Methods

### 2.1. Preparation of HL0285 Extract

The aerial parts of *Nicandra physalodes* were collected from Dali, China, shade-dried and crushed to 30 mesh. After extraction with a 3-fold volume of 75% ethyl alcohol under reflux for 2 h, the whole filtrate was concentrated by rotary evaporation, frozen for 24 h at -80°C, and dried in a vacuum freeze dryer for 72 h. The samples were packed separately and stored at -80°C for subsequent analysis and experiments.

### 2.2. Caenorhabditis elegans Maintenance and Strains

All strains were obtained from the Caenorhabditis Genetics Center (CGC) and cultured on nematode growth medium (NGM) agar plates at 16°C or 20°C. The *C. elegans* strain backgrounds were as follows: N2 *C. elegans* wild type, CF1038 *daf-16(mu86) I*, TJ356 *zIs356 [daf-16p::daf-16a/b::GFP+rol-6(su1006)]*, CF1553 *muIs84 [(pAD76) sod-3p::GFP+rol-6(su1006)]*, CL2166 *dvIs19 [(pAF15)gst-4p::GFP::NLS] III*, PS3551 *hsf-1(sy441) I*, SJ4100 *zcIs13 [hsp-6p::GFP + lin-15(+)]*, CL2070 *dvIs70 [hsp-16.2p::GFP+rol-6(su1006)]*, CL4176 *dvIs27 [myo-3p::A-Beta (1-42)::let-851 3'UTR)+rol-6(su1006)] X*, and AM140 *rmIs132 [unc-54p::Q35::YFP]*.

### 2.3. Lifespan Assay

All *C. elegans* individuals were cultured over 3 generations without starvation and subjected to lifespan experiments at 20°C. Live *Escherichia coli* (*E. coli*) strain OP50 was used as a food source, and 50 *μ*g/mL 5-fluorodeoxyuridine was added during the first 10 days to prevent the hatching of progeny. L4 stage worms were transferred to fresh NGM plates with platinum wire, and DMSO (0.5%) was used as a blank control. The number of *C. elegans* individuals surviving on each NGM plate was counted daily under a stereoscopic microscope, and the lack of movement and response to mechanical stimulus was recorded as death, excluding individuals that crawled off the plate or exhibited the vulva phenotype. Worms were transferred to fresh plates every 2 days until the end of the experiment. Experiments were independently repeated three times. Finally, the survival rate was calculated, and survival curves were plotted using GraphPad Prism 8. The log-rank (Mantel-Cox) test was used to test the statistical significance of the survival curves.

### 2.4. Bacterial Growth Assay

Bacterial growth assays were performed following the method described previously [13]. OP50 was plated onto solid LB medium and cultured inverted at 37°C overnight. In the next day, a colony was picked and inoculated into liquid LB medium, followed by culturing at 37°C for 16 h. The bacterial culture was diluted in LB medium to OD_600_ = 0.4, and 30 *μ*L of the dilution was added onto NGM plates at 20°C. The plates were rinsed with M9 buffer at 6 different time points (12 h, 24 h, 36 h, 48 h, 60 h, and 72 h) after incubation, and the OD_600_ was measured on a Hitachi U-2910 spectrometer. The experiments were repeated three times independently, and the statistical significance of the growth inhibition was assessed by multiple *t*-tests.

### 2.5. Thrashing Assay and Pharyngeal Pumping Assay

The thrashing assay and pharyngeal pumping assay were performed following the previously described methods [12]. The experiments were performed with reference to the lifespan assay. On days 3 and 8, M9 buffer was added to the blank NGM plates, and worms were acclimated for 30 s. The amount of body bending within 30 s was then determined, and a change in the direction of bending in the middle of the body was defined as thrashing [14]. Likewise, on days 3 and 8, swallowing was measured by measuring pharyngeal contractions within 30 s. The sample size for the two experiments was 20-30 worms per group. The experiments were repeated three times independently, and the statistical significance was assessed by two-way ANOVA along with Sidak multiple comparisons test.

### 2.6. Oil Red O Staining Assay

The Oil Red O staining assay was performed according to the previously described method [15]. The experiments were performed with reference to the lifespan assay. On day 3, approximately 30 worms were taken from each group for the experiment. Oil Red O solution (isopropanol, 5 mg/mL) was diluted with water (60 : 40 (vol/vol)), followed by filtration. The worms were resuspended and washed three times with M9 buffer and then transferred into 60% isopropanol to dehydrate. Then, the supernatant was removed, and 500 *μ*L of Oil Red O working solution was added with gentle shaking for 2 h in the dark. After three times of washing with M9 buffer, worms were transferred onto 3% agarose pads by platinum wire and anesthetized with 5 mg/mL levamisole. *C. elegans* images were performed using a Nikon Eclipse Ti2 microscope and quantified using ImageJ software. The sample size for the experiment was 20-30 worms per group. The experiments were repeated three times independently, and the statistical significance was assessed by unpaired *t*-test.

### 2.7. Fertility Assay

Fertility assay was performed according to the previously described method [16]. Approximately 30 worms were split into control and HL0285 group. The plates were transferred and counted daily (one worm per plate), and the daily and total egg production of worms were quantified until the end of egg-laying. The experiments were repeated three times independently, and the statistical significance was assessed by two-way ANOVA along with Sidak multiple comparisons test.

### 2.8. Aging-Related Neurodegenerative Disease Assays

Strain CL4176 was synchronized at 16°C for 36 h until the L3 larval stage and then transferred to NGM plates with or without HL0285 and cultured at 25°C. The state of the worms was observed daily with a stereoscopic microscope and recorded. Worms were considered to exhibit the pathological behavior of paralysis if they could not climb forward or backward upon light touch with platinum wire, but their heads could still swing. Finally, the number of paralyzed worms was determined, and the paralysis curves were drawn by GraphPad Prism 8. The experiment was repeated three times independently, and the statistical significance of the paralysis curve was test by log-rank (Mantel-Cox). Strain AM140 was synchronized at 20°C and grown until the L4 stage. After four days of treatment (with or without HL0285), the worms were collected and transferred onto 3% agarose pads by platinum wire and anesthetized with 5 mg/mL levamisole. Images were taken with a Nikon Eclipse Ti2 microscope and analyzed with ImageJ software. The experiments were repeated three times independently, and the statistical significance were assessed by unpaired *t*-test.

### 2.9. Stress Resistance Assays

Based on the previous experiments [17–19], stress resistance assays were performed using worms 4 days after HL0285 administration. In the oxidative stress assay, worms were transferred into 1 mL of M9 buffer containing 10 mM paraquat, with 5-6 worms per well, and the number of surviving worms was recorded every 3 h. For the heat stress resistance assay, worms were placed in a new blank NGM plate in a 35°C incubator and counted every 3 h to calculate survival percentages. For the osmotic stress resistance assay, worms were placed into NGM plates containing 500 mM NaCl. Excess M9 buffer was removed with filter paper, and the time from the dry starting point on the NGM plate was calculated. The number of crawling worms was recorded continuously on the plate with high osmotic stress for 3 min, 5 min, 7 min, 9 min, 11 min, and 13 min until all the worms were paralyzed. No movement upon touching with platinum wire was recorded as death, and these individuals were removed from the plates or wells in the stress resistance assays. The curves were finally plotted using GraphPad Prism 8. The experiments were repeated three times independently, and the statistical significance was assessed by log-rank (Mantel-Cox) tests.

### 2.10. Antioxidant Capacity Assay

The *in vitro* and *in vivo* antioxidant capacity assays of HL0285 were based on the previous studies [20, 21]. The *in vitro* antioxidant capacity was measured by a 2,2-diphenyl-1-picrylhydrazyl (DPPH) radical scavenging assay. Briefly, 100 *μ*L DPPH solution (200 *μ*M) was added to each well of a 96-well plate in advance, and then, 100 *μ*L of different concentrations of HL0285 was added separately. Ethanol was used as a negative control. The free radical clearance rate was calculated from the absorbance at 517 nm using the equation [(*A*0 − *A*1)/*A*0] × 100, where *A*0 is the absorbance of the control and *A*1 is the absorbance of HL0285. The *in vivo* antioxidant capacity was determined by measuring the level of reactive oxygen species (ROS) in *C. elegans*. After 4 days of HL0285 administration, approximately 2000 worms were collected in M9 buffer and washed 3 times with PBS until the bacteria were removed. The worm samples were then transferred to new EP tubes, snap-frozen in liquid nitrogen, and then slowly thawed at room temperature. After sonication (4°C, 6 min and 30 s), the worm samples were centrifuged (13000 rpm, 30 min, 4°C), and supernatants were collected. Each group was treated with 50 *μ*g of protein (quantified by then BCA assay, Yeasen), mixed with 200 *μ*L of DCFH-DA solution (250 *μ*M), which was used to detect ROS production, and incubated at 37°C for 1 h. The fluorescence intensity (proportional to the ROS level) was measured by a fluorescence spectrophotometer. The experiments were independently repeated three times.

### 2.11. Cell Viability Assay

MRC-5 cells were provided by Stem Cell Bank, Chinese Academy of Sciences. The cells were cultured at 37°C and 5% CO_2_ in MEM supplemented with 10% fetal bovine serum, 1% penicillin/streptomycin solution, 1% gluta-max solution, 1% nonessential amino acids solution, and 1% sodium pyruvate solution. The cells were seeded in 96-well plates (8000 cells per well) in a final volume of 100 *μ*L, and cell viability was detected by CCK8 after 72 h of treatment. MEM (100 *μ*L) containing 10% CCK8 was added to each well and coincubated with cells at 37°C for 2 h. Absorbance was measured at 450 nm using a microplate reader. The experiments were repeated three times independently, and the significance of the cell viability was assessed by unpaired *t*-test.

### 2.12. Fluorescence Microscopy

After 4 days of HL0285 treatment, GFP-harboring worms (strains CF1553, CL2166, SJ4100, CL2070, and TJ356) were transferred onto 3% agarose to detect the expression of SOD-3, GST-4, HSP-6, and HSP-16.2 and the nuclear localization of DAF-16. Worms were collected and transferred onto 3% agarose pads by platinum wire and anesthetized with 5 mg/mL levamisole. Fluorescence imaging was performed using a Nikon Eclipse Ti2 microscope (4x objective and 10x ocular), and the images were analyzed using ImageJ software. The sample size for the experiments was 20-30 worms in each group. The experiments were independently repeated three times, and the statistical significance was assessed by unpaired *t*-test.

### 2.13. Quantitative Real-Time PCR

Wild-type worms were collected on day 4 after HL0285 administration, and RNA was extracted according to the manufacturer's instructions. Total RNA was extracted using a total RNA extraction kit (Omega) and reverse-transcribed using a reverse transcription kit (Yeasen). Quantitative real-time PCR analysis was performed using the Hieff UNICON® Universal Blue qPCR SYBR Green Master Mix (Yeasen). Primers for qPCR were listed in Table S1. The experiments were independently repeated three times, and the statistical significance was assessed by unpaired *t*-test.

### 2.14. Mouse Assay

C57BL/6J mice (8 weeks old, 20-25 g) were purchased from Charles River (Beijing, China) and maintained at Shanghai SLAC Laboratory Animal Center (Shanghai, China). All procedures were performed according to the National Institutes of Health guidelines. The approval number was 20211009013. Mouse assays were conducted according to the previously reported procedures [12, 17]. Metformin served as a positive control. Doxorubicin was injected at 5 mg/kg body weight on days 0 and 10, and HL0285 (1 g/kg, 2 g/kg, intragastrically) and metformin (20 mg/kg, intragastrically) were administered from days 15-37. The vehicle was saline with 20% corn oil. After mice were sacrificed on day 38, blood samples were collected and centrifuged at 1000 × *g* for 30 min at 4°C. The upper serum was sent to KingMed Diagnostics (Shanghai, China) for alanine aminotransferase (ALT) and aspartate aminotransferase (AST) measurements.

### 2.15. SA-*β*-Gal Staining Assay

The expression of SA-*β*-Gal in MRC-5 cells and mouse kidney cells was detected by an SA-*β*-Gal staining kit. The cells were seeded in 24-well plates (10000 cells per well) in a final volume of 1 mL. After 72 h of administration, the cells were washed three times with PBS, fixed at room temperature for 15 min, and stained overnight with fresh staining solution. In the next day, the cells were washed three times with PBS, fixed at room temperature, stained with DAPI, and analyzed and photographed with a Nikon Eclipse Ti2 microscope. The experiments were independently repeated three times. Mouse kidneys were embedded in optimal cutting temperature (OCT) compound, frozen over dry ice, and then cut into 20 *μ*m sections using a freezing sliding microtome. The staining steps were consistent with those used above for MRC-5 cells. Three sections per group were photographed using a Nikon Eclipse Ti2 microscope.

### 2.16. Western Blot Analysis

Proteins were extracted from mouse liver samples with RIPA lysis buffer (strong) (Yeasen) and quantified with the BCA Protein Quantification Kit (Yeasen). Equal amounts of protein (40 *μ*g/lane) were separated by 12% sodium dodecyl sulfate-polyacrylamide gel electrophoresis (SDS-PAGE) and then transferred onto 0.22 *μ*m NC membranes. The membranes were blocked in 5% skim milk in 1% TBST overnight at 4°C and then incubated with *γ*H2AX (Abcam, ab81299) and GADPH (Proteintech, 60004-1-Ig) primary antibodies, followed by a secondary antibody (Yeasen, 33201ES60/33101ES60). Finally, the protein bands were visualized using Super ECL Detection Reagent (Yeasen, 36208ES76) with a Tanon-4600SF, and image analysis was performed with ImageJ software.

### 2.17. Liquid Chromatography-Mass Spectrometry Conditions

The potential structure of HL0285 was determined by a UHPLC System (Thermo Fisher) coupled with a Q-Exactive Orbitrap mass spectrometer. The chromatographic column was a C18 column (Agilent Zorbax Eclipse Plus C18, 100 mm × 2.1 mm, 1.8 *μ*m). Mobile phase A was water containing 0.1% (*v*/*v*) formic acid, and mobile phase B was acetonitrile at a flow rate of 0.3 mL/min. The elution gradient used was as follows: 0.0-20.0 min, 10%-50% acetonitrile; 20.0-25.0 min, 50%-90% acetonitrile; 30.0-30.1 min, 90%-10% acetonitrile; and 30.01-35.0 min, 10% acetonitrile. The sample injection volume was 5 *μ*L, and the column was maintained at 35°C. The MS/MS analysis was performed in ESI positive-ion and negative-ion modes (100.00-1500.00 m/z). High-resolution mass spectrometry was carried out with a Thermo Scientific Exactive Plus mass spectrometry (Thermo Scientific), and data analysis was performed using Xcalibur software.

### 2.18. Statistical Analysis

All quantitative data are presented as mean ± SEM. Statistical analyses included log-rank (Mantel-Cox) test, two-way ANOVA along with the Sidak multiple comparisons test, and unpaired *t*-test. Data were analyzed and figures were generated using GraphPad Prism 8 Software.

## 3. Results

### 3.1. HL0285 Extends the Lifespan and Healthspan of C. elegans

To investigate its antiaging effect, we examined the effect of HL0285 on the lifespan and healthspan of *C. elegans*. First, wild-type N2 worms were treated with 100 mg/L, 200 mg/L, or 400 mg/L HL0285 to determine the optimal concentration. The results suggested that HL0285 (200 mg/L) showed optimally prolonged activity, so this concentration was selected for further studies (Figure S1a and Table S2). Lifespan assays further confirmed that 200 mg/L HL0285 exhibited significantly prolonged activity, which reached 15.75% (^∗∗∗^*P* < 0.001) (Figure 1(b) and Table S2). To exclude the interference effect of dietary restriction, we performed a bacterial growth assay and confirmed that HL0285 had no inhibitory effect on the growth of bacteria (*E. coli*, OP50), suggesting that the lifespan extension effect of HL0285 was not due to food deprivation (Figure S1b). To systematically assess the effect of HL0285 on the healthspan of *C. elegans*, we also evaluated the aging-dependent changes in the pharyngeal pumping rate and the amount of body bending. As shown in Figure 1(c), HL0285 significantly increased the pharyngeal pumping rate at day 8 (^∗∗∗^*P* < 0.001). In addition, there was no reduction in the number of body bends on day 3 and day 8 (Figure S1c). Next, we further investigated the effect of HL0285 on lipid level to assess the effect on feeding rate. The results suggested that HL0285 did not affect lipid level in *C. elegans* (Figure S1d-e). We also assessed the effect on the number of progenies and found that HL0285 did not extend the lifespan of worms by affecting reproductive capacity (Figure S1f). The results showed that HL0285 supplementation improved feeding behavior and had no adverse effect on body bending, lipid storage, and fecundity.

Studies have shown that increases in lifespan and healthspan are usually accompanied by increased stress resistance [22, 23]. Thus, we conducted stress resistance experiments to explore the effects of HL0285 on healthspan. First, HL0285 was tested for its ability to protect N2 worms from oxidative stress by exposure to the oxidative stress inducer paraquat. As illustrated in Figure 1(d), the average lifespan of the HL0285 group (56.00 h) was prolonged by 59.43% (^∗∗∗^*P* < 0.001) compared with that of the control group (35.13 h), indicating a strong antioxidant effect. Furthermore, we examined the antioxidant effect of HL0285 *in vitro* and *in vivo*. As shown in Figure S2a, HL0285 scavenged free radicals in a concentration-dependent manner (9.77-2500.00 mg/L) *in vitro*, with the scavenging rate ranging from 3.50% to 78.16%, and it also decreased ROS level *in vivo*, as shown by ROS quantification experiments (Figure S2b-S2d), indicating that it had high antioxidant capacity. Next, we found that HL0285 prolonged the survival time of worms at 35°C, and the average lifespan of the HL0285 group (15.72 h) was significantly longer than that of the control group (12.63 h) (^∗∗∗^*P* < 0.001), indicating enhanced resistance to heat stress (Figure 1(e)). Further investigation revealed that HL0285 significantly improved the resistance of worms to osmotic stress by 9.46% (^∗^*P* < 0.05) compared with the control group (Figure 1(f)). These results showed that HL0285 not only significantly extended lifespan but also improved healthspan in *C. elegans*.

### 3.2. HL0285 Ameliorates Cellular Senescence

**Figure 2:**
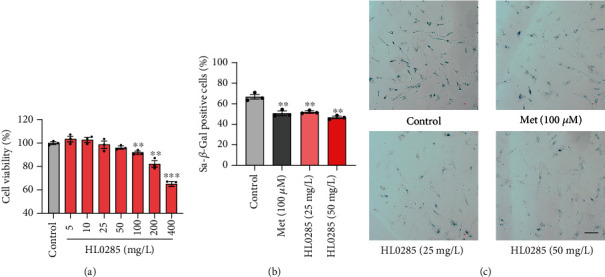
HL0285 ameliorates cellular senescence. (a) Viability of MRC-5 cells treated with HL0285 at different concentrations. (b, c) Quantification of SA-*β*-Gal positivity rate and representative images of SA-*β*-Gal staining. The concentrations of HL0285 were 25 mg/L and 50 mg/L, and 100 *μ*M Met was used as a positive control. Scale bar: 200 *μ*m. An unpaired *t*-test for (a, b) was used to calculate the *P* values. The results are presented as mean ± SEM. ^∗∗^*P* < 0.01 and ^∗∗∗^*P* < 0.001.

Since HL0285 slowed the senescence process in *C. elegans*, it was worthwhile to further explore its antiaging effect in mammalian cells. Cellular senescence is one of the hallmarks of aging and is usually accompanied by various senescence-related features [24]. SA-*β*-Gal activity is a classic biochemical marker for assessing the progress of cellular senescence [25]. In this study, MRC-5 cells, a model of replicative senescence, were used for SA-*β*-Gal staining [26]. First, the cells were treated with different concentrations of HL0285 (5-400 mg/L) for 72 h to identify the appropriate concentration for subsequent experiments. We found that the maximum nontoxic concentration of HL0285 in MRC-5 cells was 50 mg/L; thus, 25 and 50 mg/L of HL0285 were selected for subsequent experiments (Figure 2(a)). In the SA-*β*-Gal staining assay, metformin (Met) was used as a positive control, and its effective dose was described in a previous study [27]. The results showed that the average numbers of SA-*β*-Gal-positive cells in the 25 mg/L (52.03%, ^∗∗^*P* < 0.01) and 50 mg/L (46.84%, ^∗∗^*P* < 0.01) HL0285 groups were significantly lower than that in the control group (66.73%) (Figures 2(b) and 2(c)). Taken together, these results suggested that HL0285 treatment could delay cellular senescence.

### 3.3. HL0285 Counteracts Doxorubicin-Induced Aging in Mice

**Figure 3:**
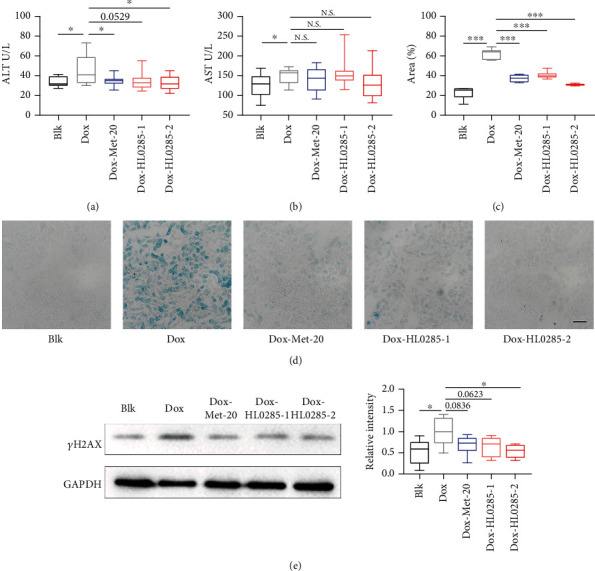
HL0285 counteracts doxorubicin-induced aging in mice. (a, b) Dox strongly increased the serum levels of ALT and AST, and HL0285 (1 g/kg, 2 g/kg) and Met (20 mg/kg, positive control) caused a reduction. (c, d) Quantification of the SA-*β*-Gal-positive rate and representative images of SA-*β*-Gal staining in frozen sections of kidney form C57BL/6J mice. Scale bar: 200 *μ*m. (e) Suppression of Dox-induced *γ*H2AX expression by HL0285 (1 g/kg, 2 g/kg) and Met (20 mg/kg, positive control). An unpaired *t*-test for (a–e) was used to calculate the *P* values. The results are presented as mean ± SEM. ^∗^*P* < 0.05 and ^∗∗∗^*P* < 0.001.

Dox is a chemotherapeutic drug that can cause DNA damage to induce aging, and this Dox-induced aging serves as a common experimental model for mammalian premature aging [28, 29]. Thus, this animal model was used further to evaluate the antiaging effect of HL0285. Dox (5 mg/kg) was applied following the procedure described in Figure S3, with Met (20 mg/kg) serving as a positive control, and the effective dose was based on a previous study [17]. As shown in Figures 3(a) and 3(b), the levels of ALT and AST, biomarkers of liver damage, were elevated after Dox treatment. HL0285 counteracted Dox-induced ALT level elevation (1 g/kg *P* = 0.0529; 2 g/kg ^∗^*P* < 0.05) and caused a slight decrease in the AST level (1 g/kg N.S.; 2 g/kg N.S.).

To further evaluate the *in vivo* antiaging effect of HL0285, we also examined the expression of senescence markers (SA-*β*-Gal) in the kidney and DNA damage markers in the liver (*γ*H2AX). The kidney SA-*β*-Gal-positive staining percentage decreased significantly after HL0285 treatment (1 g/kg ^∗∗∗^*P* < 0.001; 2 g/kg ^∗∗∗^*P* < 0.001), exhibiting levels comparable to those observed the positive control metformin (^∗∗∗^*P* < 0.001) (Figures 3(c) and 3(d)). Moreover, the results showed that HL0285 reduced the level of the protein *γ*H2AX in the liver (1 g/kg *P* < 0.0623; 2 g/kg ^∗^*P* < 0.05) (Figure 3(e)). Together, these results indicated that HL0285 could effectively counteract Dox-induced senescence accompanied by an increase in ALT, SA-*β*-Gal, and *γ*H2AX protein levels, exhibiting significant antiaging effects *in vivo*.

### 3.4. DAF-16 and HSF-1 Are Required for HL0285-Mediated Lifespan Extension in C. elegans

**Figure 4:**
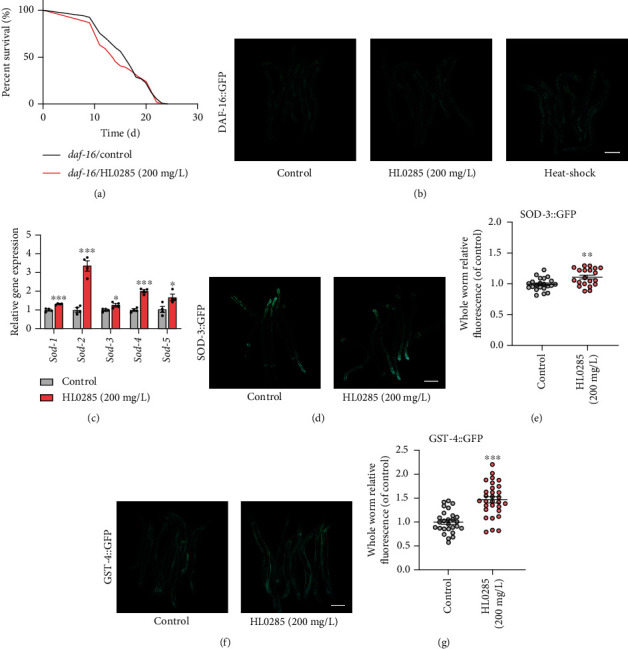
DAF-16 is required for HL0285-mediated lifespan extension in worms. (a) Survival curves of the *daf-16* mutant with HL0285 at 200 mg/L. (b) Effect of HL0285 (200 mg/L) on DAF-16 nuclear localization after 4 days of HL0285 treatment. (c) HL0285 upregulated the *daf-16*-targeted genes (*sod-1*, *sod-2*, *sod-3*, *sod-4*, and *sod-5*). Representative fluorescence images and quantitation of the expression of GFP-tagged SOD-3 (d, e) and GST-4 (f, g). Scale bar: 200 *μ*m. The log-rank (Mantel-Cox) test (a) and unpaired *t*-test (c, e, g) were used to calculate the *P* values. The results are presented as mean ± SEM. ^∗^*P* < 0.05, ^∗∗^*P* < 0.01, and ^∗∗∗^*P* < 0.001.

We previously found that HL0285 conferred high resistance to oxidative stress (Figure 1(d) and Figure S2a-S2b). Therefore, we wondered whether HL0285-mediated lifespan extension was associated with classic stress resistance-related longevity pathways, such as the insulin signaling pathway. DAF-16 is an important transcription factor downstream of the insulin signaling pathway that plays an important role in controlling longevity and stress resistance [30]. We found that HL0285 failed to extend the lifespan of the *daf-16* mutant (Figure 4(a) and Table S2), which suggested that the lifespan extension effect of HL0285 was dependent on DAF-16, even though we did not observe significant nuclear localization of DAF-16 (Figure 4(b)). We also found that expression of *sod* family genes (*sod-1*, *sod-2*, *sod-3*, *sod-4*, and *sod-5*), which are downstream of DAF-16, increased significantly (^∗^*P* < 0.05 and ^∗∗∗^*P* < 0.001) (Figure 4(c)). HL0285 treatment increased the expression of SOD-3 (^∗∗^*P* < 0.01) (Figures 4(d) and 4(e)). Moreover, the protein level of GST-4 (^∗∗∗^*P* < 0.001), another gene downstream of DAF-16, increased with HL0285 treatment (Figures 4(f) and 4(g)). Our data suggested that HL0285 extended lifespan by activating DAF-16 and upregulating the downstream related antioxidant genes.

**Figure 5:**
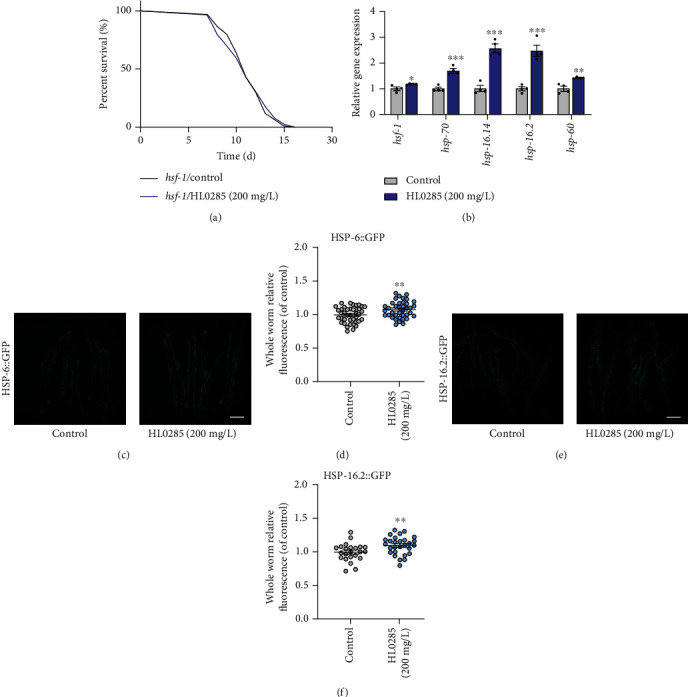
HSF-1 is required for HL0285-mediated lifespan extension in worms. (a) Survival curves of the *hsf-1* mutant with HL0285 at 200 mg/L. (b) HL0285 upregulated *hsf-1* and its downstream genes (*hsp-70*, *hsp-16.41*, *hsp-16.2*, and *hsp-60*). Representative fluorescence images and quantitation of the expression of GFP-tagged HSP-6 (c, d) and HSP-16.2 (e, f). Scale bar: 200 *μ*m. The log-rank (Mantel-Cox) test (a) and unpaired *t*-test (b, d, f) were used to calculate the *P* values. The results are presented as mean ± SEM. ^∗^*P* < 0.05, ^∗∗^*P* < 0.01, and ^∗∗∗^*P* < 0.001.

HSF-1 is also an important transcription factor in the insulin signaling pathway, especially in the regulation of resistance to heat stress. Previously, we found that HL0285 enhanced the resistance to heat stress in *C. elegans* (Figure 1(e)). Therefore, we performed related experiments to identify whether the longevity of HL0285 was dependent on HSF-1. Similarly, we found that HL0285 could not extend the lifespan of the *hsf-1* mutant (Figure 5(a) and Table S2). Moreover, qPCR assays showed that the expression of *hsf-1* and its downstream genes (*hsp-70*, *hsp-16.41*, *hsp-16.2*, and *hsp-60*) (^∗^*P* < 0.05, ^∗∗^*P* < 0.01, and ^∗∗∗^*P* < 0.001) increased significantly (Figure 5(b)). Further experiment also showed that HL0285 enhanced the expression of HSP-6 (^∗∗^*P* < 0.01) (Figures 5(c) and 5(d)) and HSP-16.2 (^∗∗^*P* < 0.01) (Figures 5(e) and 5(f)), which are downstream of HSF-1. These data also suggested that a potential mechanism underlying HL0285-mediated lifespan extension is the activation of HSF-1 and the upregulation of related downstream genes. Taken together, the above data showed that HSF-1 was required for HL0285-mediated lifespan extension in worms.

### 3.5. HL0285 Delays the Progression of Aging-Related Diseases

**Figure 6:**
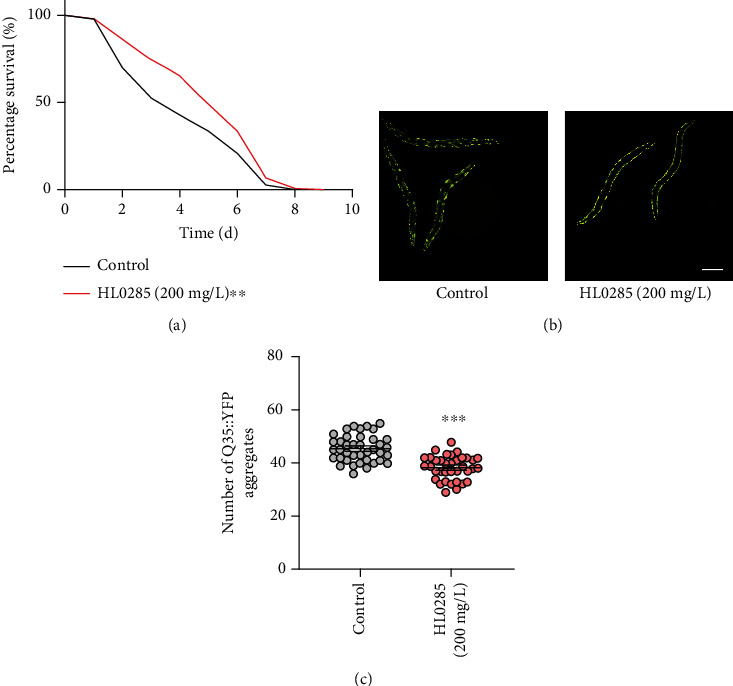
HL0285 delays the progression of aging-related diseases (a) Paralysis rate of the CL4176 strain with HL0285 at 200 mg/L. (b, c) Effect of HL0285 (200 mg/L) on the accumulation of polyQ in AM140 on day 4. Scale bar: 200 *μ*m. The log-rank (Mantel-Cox) test (a) and unpaired *t*-test (c) were used to calculate the *P* values. The results are presented as mean ± SEM. ^∗∗^*P* < 0.01 and ^∗∗∗^*P* < 0.001.

Given the better antiaging activity of HL0285, we further explored its effects in aging-related diseases, such as abnormal protein aggregation leading to neurodegenerative diseases (e.g., Alzheimer's disease (AD) and Huntington's disease (HD)). In AD, one of the most distinct clinical pathological features is A*β* (amyloid *β*-protein) deposition [31]. We employed a transgenic *C. elegans* strain (CL4176), a temperature-sensitive *C. elegans* strain overexpressing A*β* in body wall muscle cells, to evaluate the neuroprotective effect of HL0285. After 4 days of administration, a significant delay in paralysis was found in the HL0285 group (22.38%) compared with the control group, indicating a neuroprotective function against A*β*-induced toxicity (Figure 6(a), ^∗∗^*P* < 0.01). Next, we evaluated whether HL0285 has a similar positive effect in HD. Deterioration protein homeostasis, resulting from the accumulation of protein polyglutamine (polyQ) aggregates, is a hallmark of HD [32]. We measured the effect of HL0285 on the puncta aggregation of AM140, a transgenic *C. elegans* strain that expresses a polyQ::YFP fusion in body wall muscle cells. We found a significant decrease in polyQ aggregation (of approximately 15.8%) after 4 days of HL0285 treatment (^∗∗∗^*P* < 0.001) (Figures 6(b) and 6(c)). Overall, HL0285 improved the phenotype of worms in neurodegenerative disease models.

## 4. Discussion and Conclusions

*Nicandra physalodes* is a traditional medicinal plant with multiple pharmacological activities such as hypoglycemic, antioxidant, and antiparkinsonian effects [33–35]. Moreover, due to the rich content of edible pectin, the seed of *Nicandra physalodes* is often used to make snacks. From 836 kinds of plant extracts, we screened out *Nicandra physalodes* extract (HL0285) as having strong lifespan extension activity. We then immediately analyzed the effects of HL0285 on lifespan and healthspan in *C. elegans*, including on the pharyngeal pumping rate, body bending, lipid storage, and fecundity, and found that it could prolong lifespan and increase healthspan. We further demonstrated through stress resistance experiments (antioxidant, heat stress, and osmotic resistance assays) that HL0285 improved the healthspan of *C. elegans* (Figure 1). HL0285 reduced SA-*β*-Gal levels and ameliorated senescence in MRC-5 cells (Figure 2). In addition, we also used a Dox-induced premature senescence model to evaluate the antiaging effect of HL0285. Dox is a well-known chemotherapeutic agent frequently accompanied with hepatic toxicity. The mechanism of the induction senescence is mainly due to the inhibition of DNA topoisomerase, which leads to DNA damage and triggers genomic instability [36–38]. Many studies have demonstrated that Dox-induced premature is manifested by a significant increase in senescence markers such as p21^WAF1/CIP1^, IL-6, *γ*-H2AX, and SA-*β*-Gal, accompanied by an increase in liver function indicators ALT and AST [29]. In this study, we found that HL0285 counteracted the upregulation of ALT, SA-*β*-Gal, and *γ*H2AX in C57BL/6J mice with Dox-induced premature senescence (Figure 3). In short, our results indicate that HL0285 exerts its antiaging effects by activating DNA damage repair pathways to counteract DNA damage and alleviate Dox-induced premature, these results showed that the antiaging effect of HL0285 is a conserved feature.

The insulin signaling pathway is a classic longevity-related signaling pathway, and DAF-16 is a crucial transcription factor that regulates oxidative stress tolerance and prolongs lifespan [39, 40]. In this study, we found that HL0285-mediated lifespan extension was dependent on DAF-16, so HL0285 upregulated the levels of the genes *sod-1*, *sod-2*, *sod-3*, *sod-4*, and *sod-5* and the proteins SOD-3 and GST-4, even though it did not promote the nuclear localization of DAF-16, which was consistent with the previous reports [41, 42] (Figure 4). These results suggested that antioxidant effect was required for the lifespan extension effect of HL0285. HSF-1 is also an important transcription factor related to the insulin signaling pathway, especially for enhancing resistance to heat stress, inhibiting proteotoxicity, and regulating the expression of heat shock proteins [43]. In the present study, we found that HL0285 no longer showed a longevity effect in the *hsf-1* mutant; significantly upregulated the levels of *hsf-1* and the downstream genes *hsp-70*, *hsp-16.41*, *hsp-16.2*, and *hsp-60*; and enhanced the expression of HSF-1 downstream proteins HSP-6 and HSP-16.2 (Figure 5). This indicated that the antiaging effect of HL0285 was related to heat resistance to some extent. In conclusion, DAF-16 and HSF-1, two key transcription factors that regulate stress resistance against oxidation and heat, were necessary for HL0285 to exert its antiaging effects.

Research has shown that disease-associated protein aggregation is a common cause of neurodegenerative diseases [44]. In this work, we found that HL0285 delayed the progression of aging-related neurodegenerative diseases in worms by models of with AD or PD (Figure 6). Specifically, HL0285 delayed the rate of paralysis in the AD model to protect against A*β*-induced toxicity and improved protein homeostasis in the HD model by reducing polyQ accumulation. HL0285-regulated protein homeostasis in *C. elegans* is probably mediated by HSF-1 [45], but the molecular mechanism associated with its antiaging effect needs to be further investigated. In addition, the components in HL0285 were analyzed by UPLC-MS/MS. The results showed that HL0285 contains several withanolide compounds, such as nicandrenone 12, nicandrenone 17, and nicaphysalin S (Table S3), which is consistent with the previous reports [46–50]. Withanolide compounds have multiple pharmacological activities against cancer, inflammation, and cognitive dysfunction [51]. Therefore, it will be interesting to investigate whether HL0285 has antiaging effects through withanolide compounds in the future.

In conclusion, we found that HL0285 could prolong lifespan and healthspan in *C. elegans*, ameliorate cellular senescence in MRC-5 cells, and counteract premature aging in Dox-induced aging mice. Strikingly, it delayed the progression of neurodegenerative diseases in *C. elegans*. Overall, this study provides a basis for the development of *Nicandra physalodes* products.

## Data Availability

The data used and analyzed in this study are available from the corresponding author on reasonable request.

## Supplementary Materials

Supplementary Materials Table S1: primers for specific genes. Table S2: lifespan data. Figure S1: effects of HL0285 in *C. elegans* and *E. coli* OP50. Figure S2: HL0285 decreases oxidative stress in *C. elegans*. Figure S3: experimental design for Dox-induced senescence in mice. Table S3: UPLC-MS/MS putative compounds from *Nicandra physalodes*.
