# Supplementary material for: Nicandra physalodes Extract Exerts Antiaging Effects in Multiple Models and Extends the Lifespan of Caenorhabditis elegans via DAF-16 and HSF-1
Source: Oxid Med Cell Longev. 2022 Oct 11;2022:3151071. doi: 10.1155/2022/3151071 (PMC9578804; doi:10.1155/2022/3151071)
Supplement: Supplementary Materials — Table S1: primers for specific genes. Table S2: lifespan data. Figure S1: effects of HL0285 in C. elegans and E. coli OP50. Figure S2: HL0285 decreases oxidative stress in C. elegans. Figure S3: experimental design for Dox-induced senescence in mice. Table S3: UPLC-MS/MS putative compounds from Nicandra physalodes. [file 3151071.f1.docx]

**Oxidative Medicine and Cellular Longevity**

***Nicandra physaloides* extract exerts antiaging effects in multiple models and extends the lifespan of *Caenorhabditis elegans via* DAF-16 and HSF-1**

Jiqun Wang,^1, #^ Yunyuan Huang,^2, #^ Kaixuan Shi,^3, #^ Lingyuan Bao,^1^ Chaojiang Xiao,^3^ Tianyue Sun,^1^ Zhifan Mao,^1^ Jiali Feng,^1^ Zelan Hu,^1^ Zhenghan Guo,^3^ Jing Li,^3^ Bei Jiang,^3,^ * Wenwen Liu,^4,^ * Jian Li^1, 3, 4, 5,^ *

^1^State Key Laboratory of Bioreactor Engineering, Shanghai Frontiers Science Center of Optogenetic Techniques for Cell Metabolism, Frontiers Science Center for Materiobiology and Dynamic Chemistry, Shanghai Key Laboratory of New Drug Design, School of Pharmacy, East China University of Science and Technology, Shanghai 200237, China.

^2^Hubei Key Laboratory of Genetic Regulation and Integrative Biology, School of Life Sciences, Central China Normal University, Wuhan, Hubei 430079, China.

^3^Yunnan Key Laboratory of Screening and Research on Anti-pathogenic Plant Resources from West Yunnan, College of Pharmacy, Dali University, Dali, Yunnan 671000, China.

^4^Key Laboratory of Tropical Biological Resources of Ministry of Education, College of Pharmacy, Hainan University, Haikou, Hainan 570228, China.

^5^Clinical Medicine Scientific and Technical Innovation Center, Shanghai Tenth People’s Hospital, Tongji University School of Medicine, Shanghai 200092, China.

^#^Jiqun Wang, Yunyuan Huang and Kaixuan Shi contributed equally to this work.

Correspondence should be addressed to Jian Li, jianli@ecust.edu.cn; Wenwen Liu, wenwenliu@hainanu.edu.cn; Bei Jiang, jiangbei@dali.edu.cn.

Copyright © 2022 Jiqun Wang et al. This is an open access article distributed under the Creative Commons Attribution License, which permits unrestricted use, distribution, and reproduction in any medium, provided the original work is properly cited.

Table S1. Primers for specific genes.

| **Genes** | **Species** | **Forward Primer (5’ to 3’)** | **Reverse Primer (5’ to 3’)** |
| --- | --- | --- | --- |
| *gapdh* | *C. elegans* | GGAACTGTTACCTACGATGGAGA | AAACTCCAGTAGACTCGACAACG |
| *sod-1* |  | CGTAGGCGATCTAGGAAATGTG | TGACGAGCGTGTCGGTGAG |
| *sod-2* |  | GATACTGTCCAAAGGGAAAGAT | GTAGTAAGCGTGCTCCCAGA |
| *sod-3* |  | ATCTACTGCTCGCACTGCTT | TTTCATGGCTGATTACAGGTT |
| *sod-4* |  | GCACCAGATGACTCGAACA | GTCCACTTAATGAGGCAAGA |
| *sod-5* |  | TCGAAACGTGCTGTAGCGG | CACCTTCGGCTTTCTGGGT |
| *hsf-1* |  | TGTGCTGGAAATAGACTTTTGC | ACAAACATCCTTGTATGGACCT |
| *hsp-70* |  | GATCGAATTAGCTCGCGTAATC | TCCGCTAATGTATTACGTTCCA |
| *hsp-16.41* |  | AAGTTTTCGGTTCAACTCGATG | TCCCTTCAATTTTGAGCTCTCT |
| *hsp-16.2* |  | GTACGCTATCAATCCAAGGAGA | GGAATTGATCTTCCTTGAACCG |
| *hsp-60* |  | TTGTTTTACGGCTCTTTTTCCC | ATATAGAGAAAAACGGGGTGGG |

Table S2. Lifespan data.

| **Strain** | **Genotype** | **Drug**  **treatment** | **Mean lifespan**  **（days）** | **Maximum**  **Lifespan(days)** | **Number of worms** | ***P*-Values** | |
| --- | --- | --- | --- | --- | --- | --- | --- |
| N2 | *WT* | - | 13.10 | 21 | 80 | — | |
| N2 | *WT* | HL0285  (100 mg/L) | 12.67 | 24 | 79 | 0.521 |  |
| N2 | *WT* | HL0285  (200 mg/L) | 14.13 | 23 | 76 | 0.052 |  |
| N2 | *WT* | HL0285  (400 mg/L) | 13.99 | 22 | 79 | 0.076 |  |
| N2 | *WT* | - | 12.57 | 22 | 90 | — | |
| N2 | *WT* | HL0285  (200 mg/L) | 14.55 | 23 | 88 | <0.001 |  |
| N2 | *WT* | - | 12.63 | 24 | 99 | — | |
| N2 | *WT* | HL0285  (200 mg/L) | 15.46 | 23 | 105 | <0.001 |  |
| N2 | *WT* | - | 15.68 | 23 | 90 | — | |
| N2 | *WT* | HL0285  (200 mg/L) | 17.66 | 23 | 83 | <0.01 |  |
| CF1038 | *daf-16(mu86) I.* | - | 15.64 | 24 | 116 | — |  |
| CF1038 | *daf-16(mu86) I.* | HL0285  (200 mg/L) | 14.79 | 23 | 115 | 0.248 |  |
| PS3551 | *hsf-1(sy441) I.* | - | 11.19 | 16 | 119 | — |  |
| PS3551 | *hsf-1(sy441) I.* | HL0285  (200 mg/L) | 11.03 | 16 | 119 | 0.830 |  |


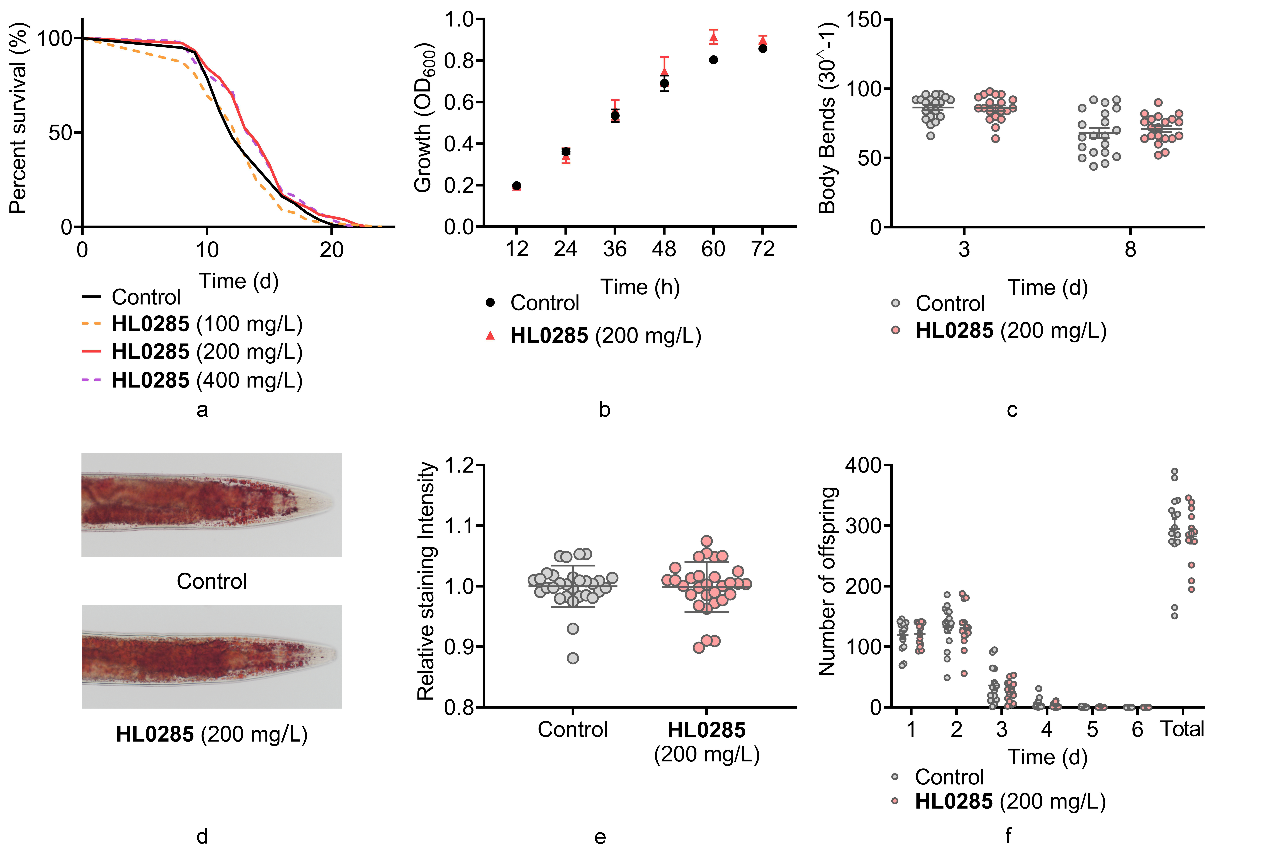
Figure S1. Effects of HL0285 in *C. elegans* and *E. coli* OP50. (a) The survival curves of *C. elegans* (wild-type) with HL0285 at 100 mg/L, 200 mg/L and 400 mg/L. (b) HL0285 did not affect growth of *E. coli* OP50. (c) The effect of HL0285 on body bends on day 3 and day 8. (d-e) Representative images and quantitative analysis of Oil Red O staining for control group and HL0285 group. (f) The effect of HL0285 on worm fecundity. Log-rank (Mantel-Cox) test (a), multiple t-tests (b), Two-way ANOVA along with Sidak multiple comparisons test (c, f) and unpaired t-test (e) were used to calculate the *P* values. The results are presented as the mean ± SEM.


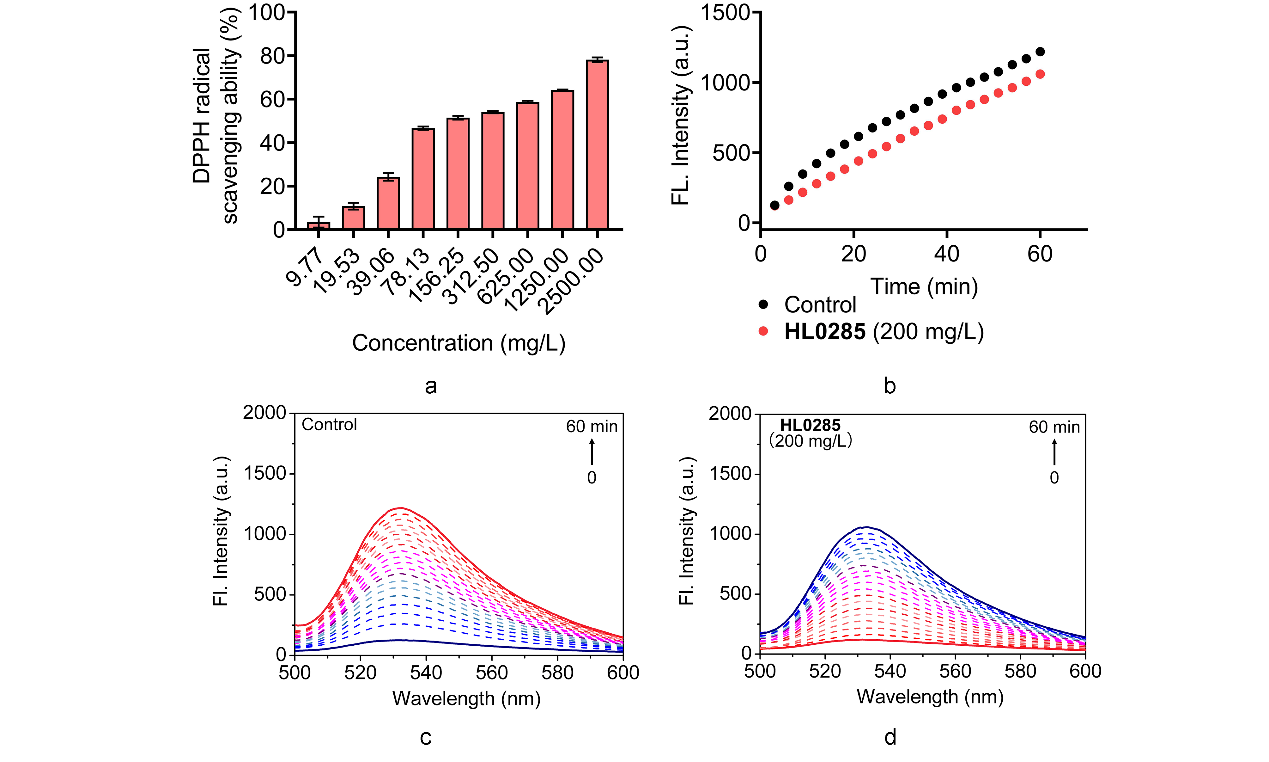
Figure S2. HL0285 decreases oxidative stress in *C. elegans*. (a) The DPPH radical scavenging ability of HL0285. (b-d) Four-day HL0285 treatment decreased ROS levels in worm lysis detected by DCFH-DA.


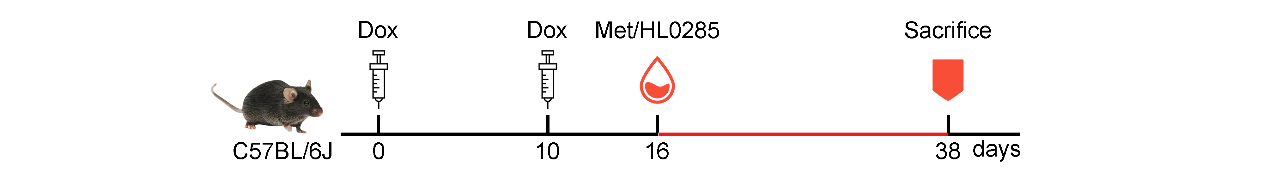
Figure S3. Experimental design for Dox-induced senescence in mice.

Table S3. UPLC-MS/MS putative compounds from *Nicandra physaloides*.

| **Retention**  **time (min)** | **Adducts** | **Exact Mass** | | **Elemental**  **Composition** | **Error**  **(ppm)** | **Putative Identity** |
| --- | --- | --- | --- | --- | --- | --- |
|  |  | Calculated (m/z) | Observed (m/z) |  |  |  |
| 1.19 | [M+Na]^+^ | 191.0318 | 191.0314 | C_8_H_8_O_4_ | 2.09 | vanillic acid |
| 10.86 | [M+H]^+^ | 411.2148 | 411.2166 | C_25_H_30_O_5_ | -4.38 | nicandrenone 17 |
| 11.3 | [M+H]^+^ | 505.2785 | 505.2795 | C_28_H_40_O_8_ | -1.98 | nicaphysalin S |
| 12.45 | [M+H]^+^ | 503.2638 | 503.2639 | C_28_H_38_O_8_ | -0.2 | nicaphysalin U |
| 14.33 | [M+H]^+^ | 521.2739 | 521.2745 | C_28_H_40_O_9_ | -1.15 | ixocarpalactone L |
| 19.92 | [M+Na]^+^ | 523.265 | 523.2666 | C_29_H_40_O_7_ | -3.06 | Nicanlode J |
| 21.87 | [M+Na]^+^ | 375.1936 | 375.193 | C_23_H_28_O_3_ | 1.6 | nic-physatone S |
| 23.48 | [M+H]^+^ | 379.1506 | 379.1515 | C_21_H_24_O_5_ | -2.37 | 6β,7α-dihydroxynicandrenone 10 |
| 24.64 | [M+Na]^+^ | 608.2642 | 608.2618 | C_35_H_39_NO_7_ | 3.95 | nicanlodes A |
| 26.7 | [M+H]^+^ | 491.3007 | 491.3003 | C_28_H_42_O_7_ | 0.81 | larnaxolida A |
| 27.18 | [M+Na]^+^ | 527.298 | 527.2979 | C_29_H_44_O_7_ | 0.19 | nicandrenone 11 |
| 27.79 | [M+Na]^+^ | 409.1975 | 409.1985 | C_23_H_30_O_5_ | -2.44 | nic-physatone J |
| 28.86 | [M+Na]^+^ | 411.2144 | 411.2141 | C_23_H_32_O_5_ | 0.73 | nic-physatone I |
| 29.64 | [M+Na]^+^ | 249.0877 | 249.0886 | C_15_H_14_O_2_ | -3.61 | pinosylvin monomethyl ether |
| 29.87 | [M+H]^+^ | 367.1888 | 367.1903 | C_23_H_26_O_4_ | -4.09 | 17-(1α/1β-Methylpropanone)-nicandrenone-10 |
| 30.99 | [M+Na]^+^ | 279.0637 | 279.0627 | C_15_H_12_O_4_ | 3.58 | 2*S*- pinocembrin |
| 32.44 | [M+Na]^+^ | 415.1875 | 415.1879 | C_25_H_28_O_4_ | -0.96 | nicandrenone 12 |
